# Acute transverse myelitis of the cervical spine secondary to psoas abscess

**DOI:** 10.1186/s12879-016-1922-3

**Published:** 2016-10-18

**Authors:** Hongyu He, Lirong Jin, Minjie Ju, Guowei Tu, Zhe Luo

**Affiliations:** 1 Department of Intensive Care Medicine, Zhongshan Hospital, Fudan University, Shanghai, China; 2 Department of Intensive Care Medicine, Floor 4, Building A, No 180, Fenglin Road, Shanghai, China; 3 Department of Neurology, Zhongshan Hospital, Fudan University, Shanghai, China

## Abstract

**Background:**

Acute transverse myelitis is uncommon and presumably results from an autoimmune process or a preceding infection. Most cases of bacterial myelitis are due to hematogenous dissemination from urinary or respiratory tract infections or contiguous spreading from a neighboring infected structure. A psoas abscess rarely spreads to higher levels of the spinal cord. No cases of acute cervical myelitis due to a psoas abscess have been previously reported.

**Case presentation:**

A 34-year-old man was transferred to our hospital due to progressive muscle weakness, sensory deficits and severe hypotension. Two weeks prior to admission, he had received low back injection to relieve back pain in a healthcare clinic. One day prior to admission, his condition had worsened. On admission, he was tetraplegic with absence of sensation below the level of the suprasternal fossa. A lumbar CT scan demonstrated an abscess in the left psoas, and the magnetic resonance imaging (MRI) scan of the entire spinal suggested a cervical spine infection. A cerebrospinal fluid (CSF) analysis performed before surgery indicated the possibility of bacterial infection. An operation was performed to drain the abscess. Microbiological cultivation revealed a Methicillin-resistant Staphylococcus aureus (MRSA) infection. The patient was administered with vancomycin for 10 days and followed by oral formulations of linezolid for 6 weeks. The patient's general condition improved, and he was successfully discharged. Six months later, a follow-up MRI revealed that the lesion of the cervical spine had been ameliorated, and the sensation and myodynamia of his upper limbs had partially recovered.

**Conclusion:**

This was a rare case of a high-level cervical spine pyogenic infection complicating psoas abscess. An invasive paravertebral injection procedure was thought to be the initial damaging event that created a port of entry for MRSA into the psoas muscle and caused a subsequent psoas abscess. This case indicated that evaluation of higher levels of the spine is warranted when a psoas abscess coexists with severe weakness.

## Background

Acute transverse myelitis (ATM) has an incidence of one to four new cases per million people per year, affecting individuals of all ages. The causes of transverse myelopathy include infectious etiology, cord compression (tumor, epidural abscess, or epidural hematoma), connective tissue disease (systemic lupus erythematosus, mixed connective tissue disease, scleroderma, ankylosing spondylitis, or rheumatoid arthritis), multiple sclerosis (MS), neuromyelitis optica, or idiopathic transverse myelitis [1, 2]. Infectious myelitis may be caused by a variety of viral, bacterial, fungal, or parasitic agents. *Mycoplasma* pneumoniae is the most common pathogen among the bacterial agents. Other bacterial infections may cause myelitis as a result of compression or ischemia due to the accumulation of purulent exudate in the subarachnoid space (*Streptococcus* pneumoniae and *Staphylococcus aureus*) or through an immune-mediated reaction (*Rickettsiae*, *Chlamydophila,* and *Bartonella henselae*). Infectious granulomatous diseases such as tuberculosis*,* syphilis, and brucellosis may also contribute to bacterial myelitis [3, 4]. Most cases of transverse myelitis are idiopathic and presumably result from an autoimmune process, and up to half of these patients have a preceding infection. Most infectious myelitis results from hematogenous dissemination from a remote focus such as a urinary or respiratory tract infection or by contiguous spread from a neighboring infected structure such as vertebral osteomyelitis or a paraspinal abscess [5]. No cases of cervical myelitis due to a psoas abscess have been reported. Thus, we report the first case of acute paralysis due to a cervical spinal cord infection resulting from a psoas abscess.

## Case presentation

**Fig. 1:**
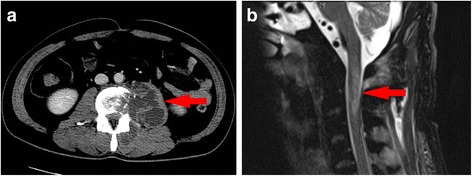
A psoas abscess and its remote involvement. **a** Ring enhancement in the left psoas muscle on an enhanced CT image obtained on the day of admission. **b** The cervical MRI findings revealed altered signal intensity involving C2-3, with cord expansion on T2-weighted sagittal sections, suggestive of myelitis

A 34-year-old man complained of back pain for 2 weeks and then received lower back acupuncture and paravertebral injections to relieve the pain in a healthcare clinic. The pain was not ameliorated, and a mass was discovered in the left psoas. Then, the patient gradually developed a high fever, sensory deficits, progressive muscle weakness, bladder and bowel dysfunction, chest tightness, and dyspnea. The patient was transferred to our hospital. This patient had no history of HIV, syphilis or gastrointestinal surgery. The examination findings on admission were as follows: body temperature, 41 °C; blood pressure, 95/51 mmHg (on norepinephrine); pulse rate, 110 bpm; and respiratory rate, 15 bpm (on mechanical ventilation). A mass could be palpated in the lower back with local tenderness and increased local skin temperature. He was lethargic with normal cranial nerve functions. He could nod his head, and no signs of meningeal irritation or elevated intracranial tension were present. He developed tetraplegia and had Grade 0/5 limb myodynamia with generalized areflexia and hypotonia. The superficial abdominal and tendon reflexes were not elicitable. Babinski sign was negative. He could not breathe spontaneously and required mechanical ventilation. Bladder and bowel dysfunction were present. Sensory deficits were present below the level of the suprasternal fossa. Blood investigations revealed leukocytosis and elevated C-reactive protein. Serological tests for HIV and RPR were negative. The tests of B lymphocytes, T lymphocytes, CD4+, CD8+ and NK cells counts were normal. Autoantibodies including serum ANA, Ro/SSA,La/SSB antibodies, antibodies to extractable nuclear antigen, rheumatoid factor, antiphospholipid antibodies, and antineutrophil cytoplasmic antibodies were negative. The hemoglobin (Hb) level was normal and there was no evidence indicating deficiency of folic acid or VitB12. A lumbar CT evaluation revealed a hematoma in the left psoas. The MRI of spine revealed altered signal intensity involving C2-3 with cord expansion on T2-weighted suggested the diagnosis of myelitis (Fig. 1). The cranial MRI findings were normal. No evidence of thoracic and lumbar myelitis was found. The primary diagnoses were psoas muscle abscess and acute myelitis. A lumbar puncture was performed. The opening pressure was 180 mm H_2_O. The CSF was cloudy with a white blood cell count 5000 /μL, glucose 0.6 mmol/L and protein 5.0 g/L. The emergent operation was performed to drain the abscess [6]. During the operation, over 300 ml of pus in the psoas muscle was drained. Further, the abscess invaded intervertebral space. The pus, blood, and CSF cultures all revealed Methicillin-resistant Staphylococcus aureus (MRSA). The histopathological examination of lumbar vertebrae suggested nonspecific inflammation without evidence of tuberculosis infection. Therefore, systemic antibiotics were administered with vancomycin for 10 days and followed by oral formulations of linezolid for 6 weeks [7, 8].

**Fig. 2:**
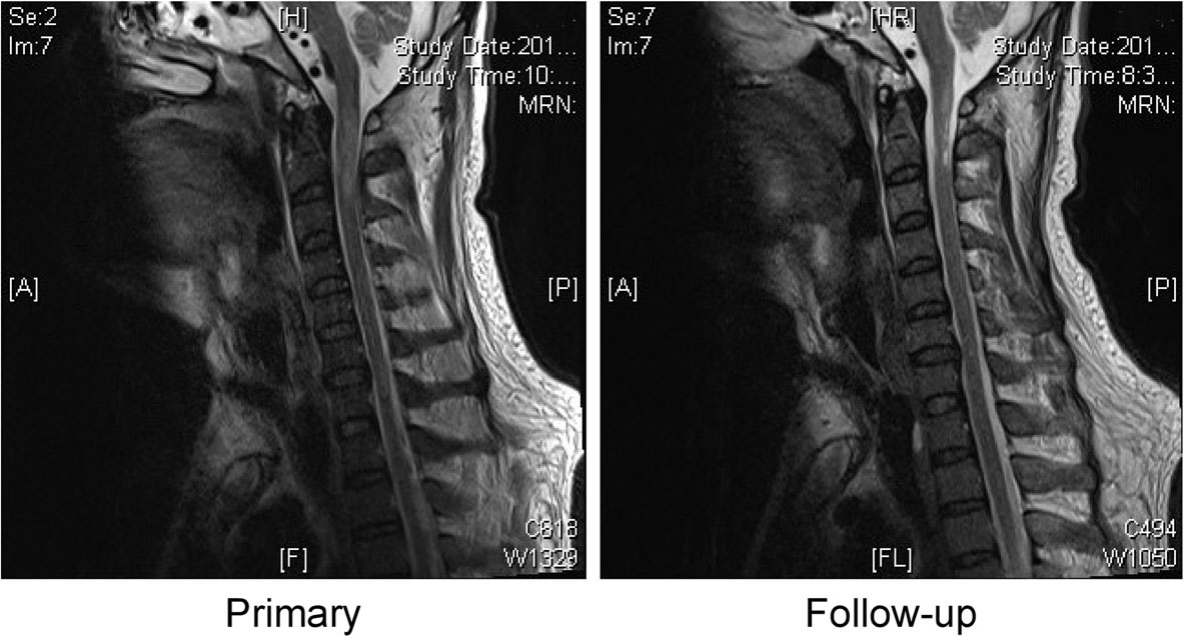
A follow-up cervical MRI evaluation revealed a less extensive hyperintense region on sagittal sections of T2-weighted images

With the administration of effective antibiotics, the patient gradually improved and showed stable vital signs 6 months later, he could move his fingers and hands, and the myodynamia of the upper limbs had increased to Grade 3/5. He had skin sensation above the T4 level and could wean from mechanical ventilator during the daytime. A follow-up MRI revealed that the lesion of the spinal cord was ameliorated (Fig. 2).

## Conclusion

Acute transverse myelitis is characterized clinically by acutely or subacutely developing symptoms and signs of neurologic dysfunction in motor, sensory, and autonomic nerves and the nerve tracts of the spinal cord [1]. The terms “acute transverse myelopathy” and “acute transverse myelitis” are frequently used interchangeably in the published literature [9]. Spinal MRI and a lumbar puncture are mandatory for the evaluation of suspected ATM. This was a rare case of infectious cervical myelitis complicating a psoas abscess. The psoas abscess was possibly caused by lower back acupuncture and paravertebral injections according to medical history. Regarding the route of infectious invasion of cervical spine, we have two speculations. The first possible interpretation was via CSF spreading. The invasive procedures created a port of entry for MRSA and led to the psoas abscess. Then, the organism penetrated the arachnoid and contaminated CSF leading to myelitis. However, it’s hard to explain why cervical spine was involved instead of lumbar spine. The second possible interpretation was hematogenous dissemination. The blood stream infection of MRSA due to the psoas abscess might cause myelitis of cervical cord. The case indicated that evaluation of higher levels of the spine should be warranted when a psoas abscess coexisted with severe motor deficits.

## Abbreviations

CSF: Cerebrospinal Fluid
CT: Computerized Tomography
MRI: Magnetic Resonance Imaging
MRSA: Methicillin Resistant Staphylococcus Aureus
